# Supplementary material for: Sustained-input switches for transcription factors and microRNAs are central building blocks of eukaryotic gene circuits
Source: Genome Biol. 2013 Aug 23;14(8):R85. doi: 10.1186/gb-2013-14-8-r85 (PMC4054853; doi:10.1186/gb-2013-14-8-r85)
Supplement: Additional file 5 — HTML Browsable Motif Output. Zipped folder containing all WaRSwap and FANMOD motif output, viewable in a web browser. [file gb-2013-14-8-r85-S5.ZIP › HTML_browsable_motif_output/FANMOD_ath_tair9/sigs_fanmodm-2000.pvals.heatmaps.html/motif_id_6_000101001_tftype_ath_upstream_-2000_0.html]

```
BG_MODEL = FANMOD
MOTIF_ID = 6_000101001
TF_TYPE = ath
UPSTREAM = -2000_0


PVals
FN_0.2	FN_0.4	FN_0.6	FN_0.8
dg_60.genes	0.698	0.865	0.735	0
dg_70.genes	0.667	0.879	0.737	0
dg_80.genes	0.663	0.851	0.699	0

ZScores
FN_0.2	FN_0.4	FN_0.6	FN_0.8
dg_60.genes	-0.514	-1.103	-0.643	3.797
dg_70.genes	-0.453	-1.163	-0.648	3.58
dg_80.genes	-0.428	-1.097	-0.55	3.727

StDevs
FN_0.2	FN_0.4	FN_0.6	FN_0.8
dg_60.genes	192.799	262.628	127.002	26.279
dg_70.genes	195.314	265.491	127.113	27.134
dg_80.genes	187.746	254.325	129.705	26.237
```
